# Femur performed better than tibia in autologous transplantation during hemipelvis reconstruction

**DOI:** 10.1186/1477-7819-12-1

**Published:** 2014-01-04

**Authors:** Jiong Mei, Ming Ni, You-Shui Gao, Zhi-Yuan Wang

**Affiliations:** 1 Department of Orthopedics, Tongji Hospital, Tongji University, Shanghai, 200065, China; 2 Department of Orthopedics, Shanghai Pudong New Area People’s Hospital, Shanghai, 201200, China; 3 Department of Orthopedics, Shanghai Sixth People’s Hospital, Shanghai JiaoTong University, Shanghai, 200233, China

**Keywords:** Biomechanics, Finite-element analysis, Hemipelvectomy, Pelvis, Reconstruction

## Abstract

**Background:**

Pelvic reconstruction after hemipelvectomy can greatly improve the weight-bearing stability of the supporting skeleton and improve patients’ quality of life. Although an autograft can be used to reconstruct pelvic defects, the most suitable choice of autograft, i.e., the use of either femur or tibia, has not been determined. We aimed to analyze the mechanical stresses of a pelvic ring reconstructed using femur or tibia after hemipelvectomy using finite element (FE) analysis.

**Methods:**

FE models of normal and reconstructed pelvis were established based on computed tomography images, and the stress distributions were analyzed under physiological loading from 0 to 500 N in both intact and restored pelvic models using femur or tibia.

**Results:**

The vertical displacement of the intact pelvis was less than that of reconstructed pelvis, but there was no significant difference between the two reconstructed models. In FE analysis, the stress distribution of the intact pelvic model was bilaterally symmetric and the maximum stresses were located at the sacroiliac joint, arcuate line, ischiatic ramus, and ischial tuberosity. The maximum stress in each part of the reconstructed pelvis greatly exceeded that of the intact model. The maximum von Mises stress of the femur was 13.9 MPa, and that of the tibia was 6.41 MPa. However, the stress distribution was different in the two types of reconstructed pelvises. The tibial reconstruction model induced concentrated stress on the tibia shaft making it more vulnerable to fracture. The maximum stress on the femur was concentrated on the connections between the femur and the screws.

**Conclusions:**

From a biomechanical point of view, the reconstruction of hemipelvic defects with femur is a better choice.

## Background

Treatment of pelvic malignancies, especially those involving the periacetabulum, continues to be one of the most challenging problems for oncological and orthopedic surgeons [1-4]. With the advancement of preoperative adjuvant treatments, improvements in imaging and surgical techniques, and the development of oncologically correct limb-salvage procedures, indications for hemipelvectomy in patients with pelvic malignancies have decreased dramatically [5-7]. However, in rare and complicated cases, amputation is still an option for this patient population [8]. Hemipelvectomy may also be life-saving for patients with severe pelvic trauma or uncontrollable sepsis of the lower extremity, and it can provide significant palliation for uncontrollable metastatic lesions of the lower extremities [9-13].

Functional outcomes and quality of life (QOL) of patients who have undergone hemipelvectomy are typically poor. One report that analyzed the QOL of patients with pelvic sarcomas who underwent acetabular resection and reconstruction by different methods found that patients who received hindquarter amputation had the lowest functional score and had to use a wheelchair or crutches in the house [14]. A report that reviewed 68 cases of hindquarter amputation found that only three patients were fitted with prostheses and had regained the ability to ambulate without external supports, and ten patients were wheelchair bound or spent most of their time in bed; patients reported significant restrictions performing the activities of daily life and leisure activities [15,16].

In patients who have undergone hemipelvectomy, the stability of the supporting skeleton for weight bearing can be increased through restoration of pelvic ring continuity [17,18]. Reconstruction of the pelvic ring with an autograft after hindquarter amputation can result in improvement in sitting stability and prosthesis support [19]. The reconstruction of the pelvic ring with an autograft from the femur or tibia has been reported [17], indicating three cases in which the hemipelvectomy defect was restored with femur or tibia, harvested from the discarded amputated limb and stripped of all soft tissue. Postoperatively, the patients’ sitting stability was excellent, and they used their prosthesis without problems. The authors postulated that in selected cases, the method could improve functional outcome and QOL. However, few studies have examined pelvic reconstruction with an autograft after hindquarter amputation.

The assessment of stress distribution throughout an entire reconstructed pelvis using mathematical models or experiments with implanted prostheses and cadaveric specimens is difficult and inaccurate. Finite-element (FE) analysis, however, can accommodate large variations in bone geometry and material properties and the technology allows the creation of FE models based on computed tomography (CT) data.

To the best of the authors’ knowledge, there has been no anatomical and mechanical analyses of pelvic reconstruction with femur or tibia after hindquarter amputation. The purpose of the present study was to analyze the mechanical stresses of a pelvic ring reconstructed with autologous femur or tibia after hemipelvectomy using FE analysis.

## Methods

### Specimens

Ten cadaveric pelvic specimens (six male, four female) along with the lumbar and bilateral lower limbs were obtained from the Anatomy Department of Tongji University Medical School. All soft tissues were dissected from the specimens, sparing the hip joint capsule and the ligaments of the pelvic ring and floor. The average age of the specimens was 56 years (range 48–71 years). All samples were checked by X-ray to exclude any gross structural abnormalities and bone diseases. This study was approved by the Institutional Review Board of Tongji University Medical School. All specimens were obtained from persons who had provided consent for their use prior to their death.

### Strain measurement

**Figure 1:**
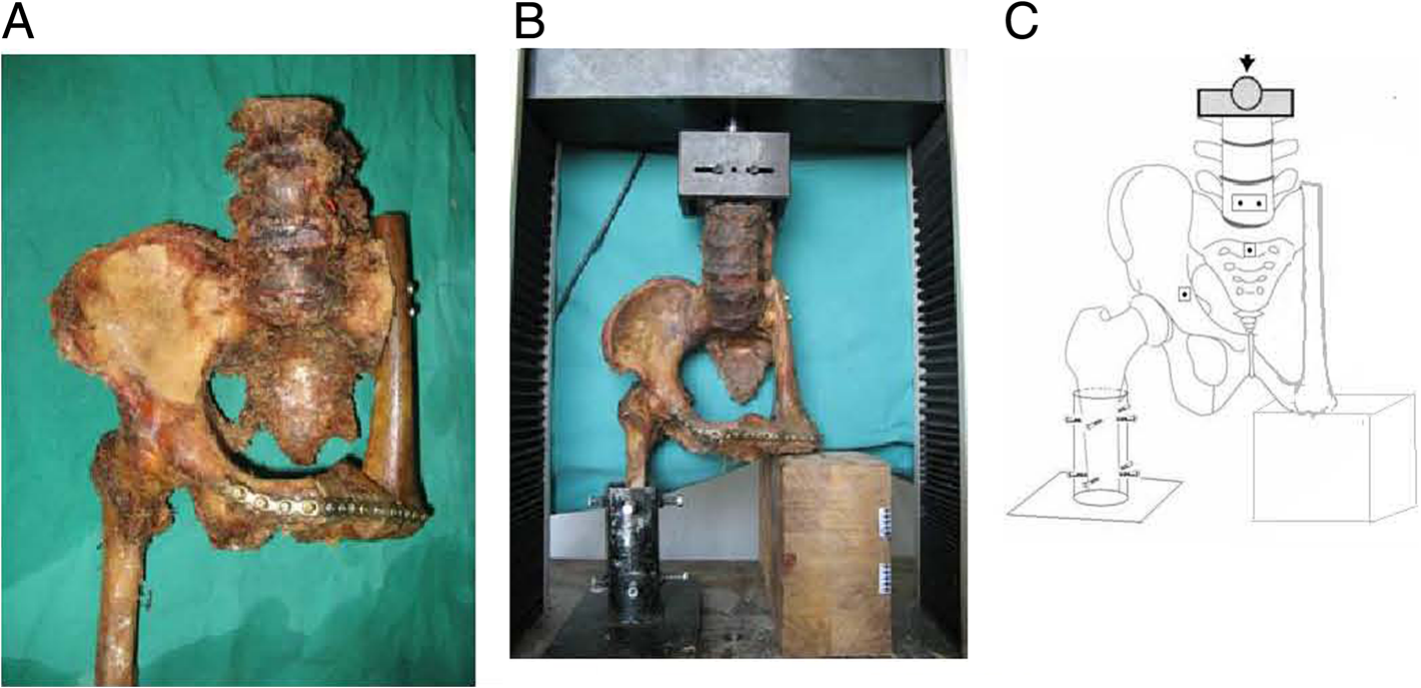
**Strain measurement process. (A)** Representative image of cadaveric hemipelvic reconstruction. **(B)** Placement of reconstructed pelvis in measurement device. **(C)** Illustration of force placement.

Models of hemipelvectomy were established in ten cadaveric specimens according to the Enneking’s Resection System [20]. Then, eight pelvises were reconstructed with autografts of the femur or tibia (four specimens each) and the ischial tuberosity was replaced by femoral condyle or tibial plateau. The defect between the symphysis pubis and reconstructed bone was filled with bridge bone. Two long lag screws were used to stabilize the proximal femur to the sacrum. Reconstructed bones were fixed to the pubic branch of the left hemipelvis with plates and screws (Figure 1A). The proximal third of both femurs of the intact pelvis was fixed with custom-made jigs which restricted movement to simulate stance on two feet. For the reconstructed pelvises, the right femur was fixed by a jig, while the reconstructed left side was supported by a wooden box (Figure 1B). Correct stance was achieved by alignment of bilateral anterior superior iliac spines and pubic symphysis in the coronal plane. An axial load, varying from 0 to 500 N, was applied to the proximal lumbar region and the force was transmitted to the upper surface of the sacrum using a pelvic testing machine (CSS-44010, Changchun Institute for Testing Machines; Jilin, China; Figure 1C). The load–displacement curves of intact (two specimens) and the eight reconstructed pelvises were established, and the stability and rigidity of the two types of pelvis were compared.

### Construction of finite element models and analysis

The FE model of the intact pelvis was constructed based on CT images of the pelvis of a healthy 30-year-old male. After the hemipelvis was removed, autograft and implants were added and the 3-dimensional (3D) reconstructed pelvic models were established. A constant cortical thickness was assumed to be 3 mm for the pelvis [21].

The material properties of the implants were assigned according to data reported previously [22]. A FE analysis was performed to account for the von Mises stress distribution on the bones and instruments in the intact and reconstructed pelvises under a vertical load of 500 N. The degree of freedom of nodes at the normal and reconstructed ischial tuberosities of the three models was constrained.

Statistical analyses of the experimental data were performed with SPSS 13.0 software (Chicago, IL, USA).

## Results

**Figure 2:**
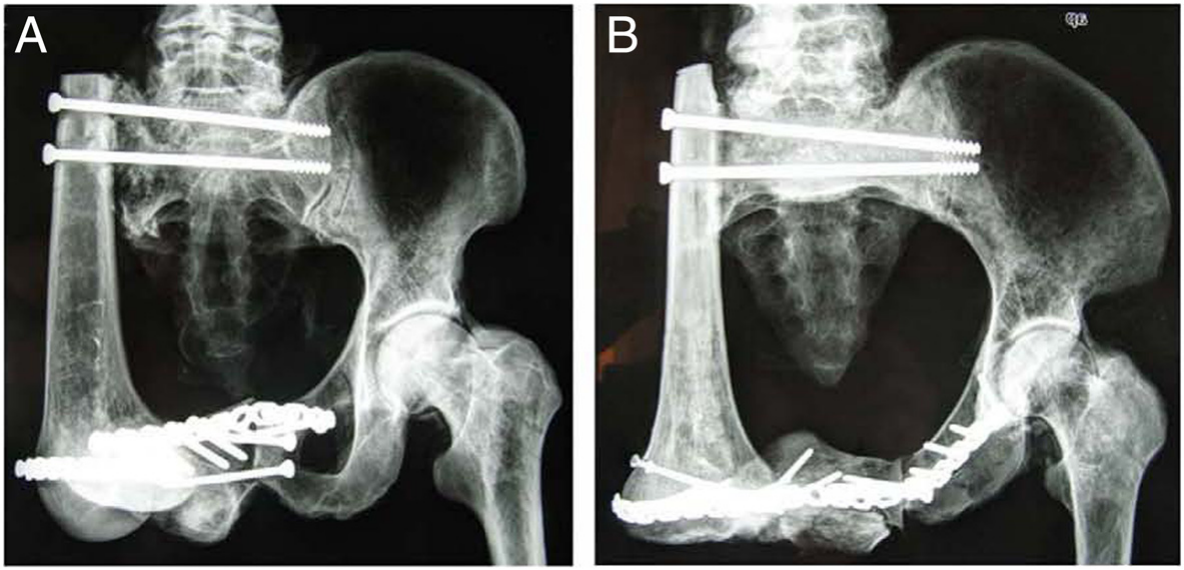
**Radiographs of the reconstructed pelvic ring.** Hemipelvic defects were stabilized with a screw-plate system. **(A)** Pelvis reconstructed with femoral autograft. **(B)** Pelvis reconstructed with tibial autograft.

Radiographs of the hemipelvic defects, reconstructed with autograft femur and tibia, are shown in Figure 2. Both the intact and reconstructed pelvises moved vertically downwards under axial load, and rebounded to their prior heights after removal of the load. This demonstrated that the reconstructive method for the pelvis was reliable. The vertical displacement of the intact pelvis was less than that of the reconstructed pelvis, but there was no significant difference between the two reconstructed models. The vertical displacement and load–displacement data are shown in Table 1.

**Table 1:** Vertical displacement of pelvis under vertical loading (mm)

	**0 N**	**100 N**	**200 N**	**300 N**	**400 N**	**500 N**
Intact pelvis	0	0.78 ± 0.41	1.12 ± 0.48	1.45 ± 0.55	1.76 ± 0.61	2.08 ± 0.67
Reconstructed by femur	0	1.07 ± 0.21	1.96 ± 0.36	2.77 ± 0.40	3.58 ± 0.36	4.38 ± 0.31
Reconstructed by tibia	0	1.12 ± 0.51	1.94 ± 0.50	2.66 ± 0.48	3.50 ± 0.42	4.30 ± 0.42

Data are presented as mean ± SD.

**Figure 3:**
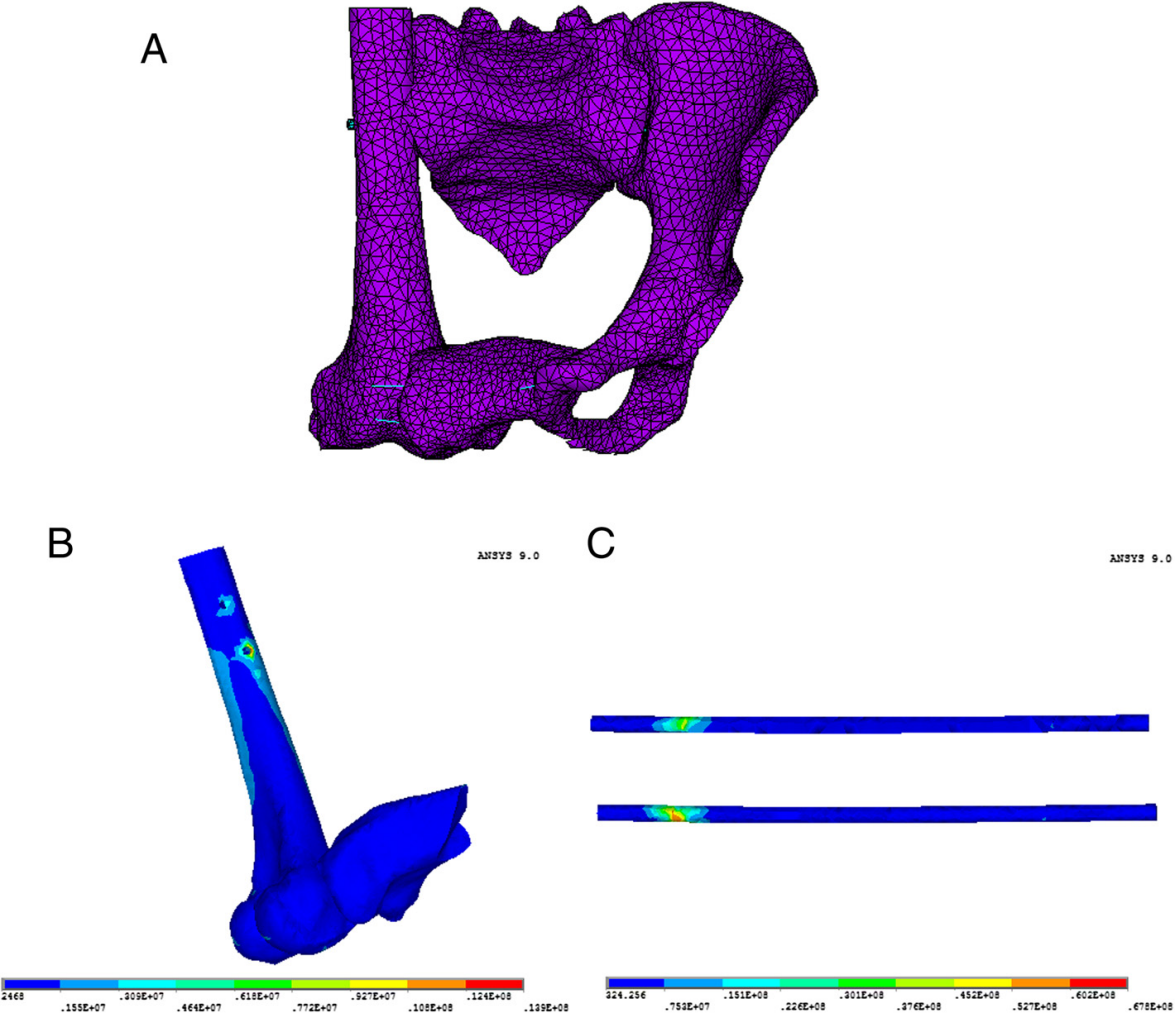
**Finite element analysis of femoral reconstructed hemipelvic ring. (A)** Finite element model of hemipelvic defect reconstructed with femoral autograft. **(B)** von Mises stress distribution on the femur. **(C)** von Mises stress distribution along the X-axis.

**Figure 4:**
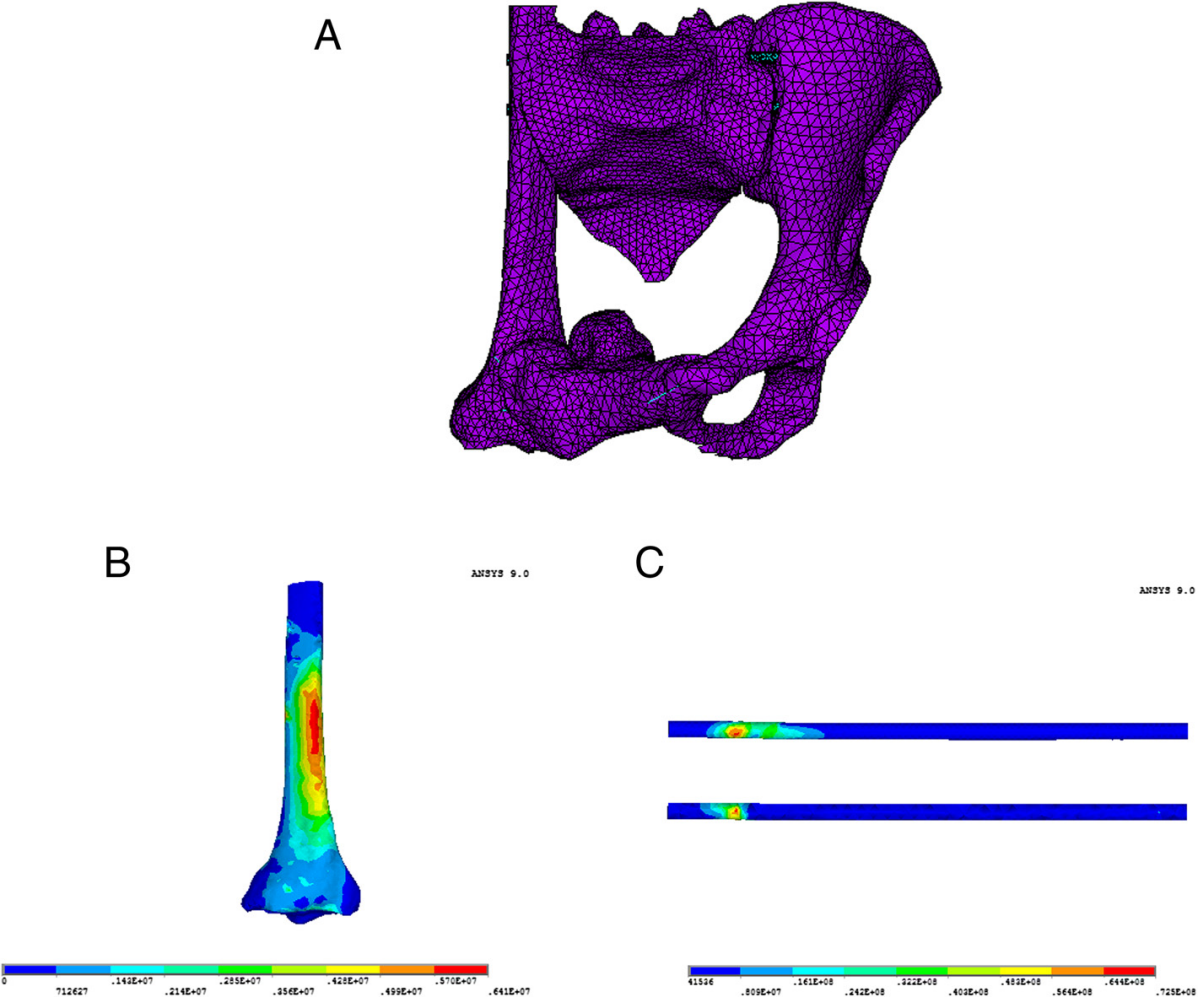
**Finite element analysis of tibial reconstructed hemipelvic ring. (A)** Finite element model of hemipelvic defect reconstructed with tibial autograft. **(B)** von Mises stress distribution on the tibia. **(C)** von Mises stress distribution along the X-axis.

In FE analysis, the stress distribution of the intact pelvis model was bilaterally symmetric, and the maximum stresses were located at sacroiliac joint (SIJ), arcuate line, ischiatic ramus, and ischial tuberosity. The maximum stress on each part of the reconstructed pelvis greatly exceeded that of the intact model; however, the stress distributions in the two types of reconstructed pelvises were similar. The maximum von Mises stress of the femur was 13.9 MPa, and that of the tibia was 6.41 MPa. The maximum stress was located on the shaft of the tibia. The stress distributions in each part of the reconstructed pelvises are shown in Table 2, and the stress distributions on the femur and tibia are shown in Figures 3 and 4, respectively.

**Table 2:** Maximum von Mises stress on each part of the pelvis (MPa)

	**Sacrum**	**Right ilium**	**Left ilium**	**Bridge bone**	**Lag screw**
Intact pelvis	5.40	6.21	5.84	–	–
Reconstructed by femur (left)	21.3	23.7	13.9	2.43	67.8
Reconstructed by tibia (left)	68.1	32.1	6.41	3.49	72.5

## Discussion

In the current study, models of pelvises reconstructed with femur and tibia after hindquarter amputation were successfully established, and FE analysis was used to examine their biomechanical properties. We analyzed the displacement of intact and reconstructed pelvises under a physiological load of 0 to 500 N, and the results demonstrated that the reconstructed pelvises had excellent stability. This implies that patients would be able to sit and walk with a prosthesis shortly after surgery if this method of reconstruction is used after hindquarter amputation. The lesser displacement of the intact pelvis under 0 to 500 N indicated that its stability was better compared with that of the reconstructed pelvises, suggesting that with this reconstruction method, full weight bearing should be avoided until bone fusion has occurred. The use of pedicled autografts for the reconstruction of pelvic defects reportedly leads to a more rapid bone union [23,24].

There are many methods that have been used to reconstruct the pelvis after hemipelvectomy that include allografting and prostheses [25-28]. Regardless of the method, restoration of pelvic ring continuity increases the stability of skeletal support for weight bearing in both sitting and standing positions and advances in reconstructive surgery have led to the ability to reconstruct pelvic ring defects. Few studies, however, have provided a detailed analysis of the stresses resulting from reconstruction of the pelvic ring. Ji et al. [29] reported a FE analysis of the reconstruction of type II + III pelvic resection with a modular hemipelvic endoprosthesis. The authors found no difference in stresses along the bilateral arcuate lines between a reconstructed pelvis and normal pelvis, but found that the stress distribution on the prosthesis along the sciatic notch was significantly greater than on the unaffected side, and that the posterior side column between the point of iliac fixation and the acetabulum was subjected to the greatest stress.

Different stress distributions were found between the intact and reconstructed pelvises in this study. An unbalanced stress distribution was found in the resected pelvis, and the higher stress distribution noted in the reconstructed pelvis was related to the stress block of the two lag screws. FE modeling showed that maximum von Mises stresses in the reconstructed pelvis were in the area of the connection of the femur and sacrum, left end of the screws, and right SIJ. These stresses could lead to degeneration of the SIJ on the normal side, development of scoliosis, and a contralateral pelvic tilt [30]. The use of larger diameter screws (7.3 or 7.5 mm) would prevent too much stress at the SIJ on the normal side. The stress concentration on the left end of the screws was related to their small contact surface with the femur, and larger screws would increase the interface with the SIJ, sacrum, and femur, thus decreasing the maximum stress on the SIJ on the normal side.

The stress distribution in the reconstructed bone was different for the two reconstructed pelvic models. In the femoral model, the von Mises stress was 13.9 MPa, whereas in the tibial model, the maximum stress at the tibia was only 6.41 MPa, significantly less than that in the femoral model and less than 80 MPa, the yield stress of cortical bone. It should be noted that in the tibial reconstructed model, the von Mises stress was concentrated on the tibial shaft, which could lead to partial or complete fracture of the tibia and reconstruction failure. From a biomechanical point of view, distal femur reconstruction of the pelvis is suitable after hindquarter amputation.

This study had several limitations. Only an axial compressive load was applied in this study, but *in vivo* forces applied to the pelvis are more complex. Further mechanical evaluation of the reconstructed pelvis regarding flexion, extension, bending, and rotation are required. Moreover, the FE models of the pelvis in the current study also need further modifications. In the current models, the SIJ was regarded as fused, which is an over simplification of the real SIJ and could lead to some deviations in the results. In addition, the model would be more accurate with the addition of elastic tissues such as ligaments and muscles.

## Conclusions

In this study, we performed FE analysis of the reconstructed hemipelvis using allograft femur and tibia, and found more stress concentration when the tibia was used for reconstruction, increasing the possibility of breakage. Thus, the femur may be a better choice for reconstruction of hemipelvic defects.

## Abbreviations

CT: Computed tomography; FE: Finite element; QOL: Quality of life; SIJ: Sacroiliac joint.

## Competing interests

All authors declare no conflict of interest.

## Authors’ contributions

JM participated in study concepts and study design; MN carried out the experimental studies; YSG participated in the literature research and carried out the data analysis; ZYW participated in the experimental studies and literature research. All authors read and approved the final manuscript.
